# Improving Firm’s Economic and Environmental Performance Through the Sustainable and Innovative Environment: Evidence From an Emerging Economy

**DOI:** 10.3389/fpsyg.2021.651394

**Published:** 2021-11-04

**Authors:** Naveed Ahmad, Miklas Scholz, Esra AlDhaen, Zia Ullah, Philippa Scholz

**Affiliations:** ^1^Faculty of Management Studies, University of Central Punjab, Lahore, Pakistan; ^2^Department of Management Sciences, Virtual University of Pakistan, Lahore, Pakistan; ^3^Division of Water Resources Engineering, Department of Building and Environmental Technology, Faculty of Engineering, Lund University, Lund, Sweden; ^4^Department of Civil Engineering Science, School of Civil Engineering and the Built Environment, University of Johannesburg, Johannesburg, South Africa; ^5^Department of Town Planning, Engineering Networks, and Systems, South Ural State University, Chelyabinsk, Russia; ^6^Institute of Environmental Engineering, Wrocław University of Environmental and Life Sciences, Wrocław, Poland; ^7^Marketing Department, College of Business and Finance, Ahlia University, Manama, Bahrain; ^8^Leads Business School, Lahore Leads University, Lahore, Pakistan; ^9^Department of Human Geography, Lund University, Lund, Sweden

**Keywords:** environmental sustainability, environmental innovation, economic performance, environmental performance, corporate social responsibility

## Abstract

Businesses in the present era are dealing with a complex and unprecedented brew of social, environmental, and technological trends. This requires sophisticated, sustainability-based management. Yet organizations are often reluctant to place sustainability core to their business strategies with the mistaken belief that the costs associated with environmental investments outweigh the benefits. The Global Climate Risk Index has placed Pakistan on 5th position in the list of nations, most susceptible to climate change in its recent report. Pakistan lost the lives of 9,989 people, incurred economic losses of $ 3.8 billion, and faced 152 shocking climates between 1999 and 2018. Based on this information, it is established that Pakistan’s susceptibility to climate change is growing unprecedentedly and industrial pollution is one of the biggest contributors in this respect. The country needs to take emergency measures to address this issue. With this background, the present study aims to investigate the impact of environmental sustainability on environmental and economic performance (EP) with the mediating effect of environmental innovation (EI) in the manufacturing sector of Pakistan. The results show that environmental sustainability is a significant predictor of environmental performance and EP and EI mediates this relationship. The findings of the present study provide better insights to policymakers to address the environmental degradation, resulting from industrial pollution.

## Introduction

Environmental sustainability (ES) is acknowledged as a crucial factor in business operations in recent days. ES is all about the practices and moves that an organization takes place in order to preserve the natural environment and resources, for example, water, air, and soil (Danso et al., 2019). In the present era, ES is regarded as a global issue that has come to the surface at an organizational level due to certain reasons such as global warming (Papalexiou and Montanari, 2019), scarcity of natural resources (Tsuboi, 2019), greenhouse gasses emission (Yusuf et al., 2020), and awareness of consumers about eco-friendly manufacturing practices of the corporations (Sarkis and Zhu, 2018; Afum et al., 2020). According to Howes et al. (2017), ES related issues are crucial for the manufacturing sector due to two principle reasons, firstly, in the present age, manufacturing organizations have to publish their data about the utilization of energy resources and the damage done to the environment due to the consumption of these resources. Secondly, in many countries, there are laws and legal regulations which ensure the quality impact of business practices on overall society. The basic reason for all that lies in the fact that the manufacturing sector contains the largest number of employees on one hand and impacts the external community on the other hand. Hence the manufacturing sector is held accountable for conducting business activities in an environment that is healthy and least disastrous to nature (Singh, 2019). Jaca et al. (2018) contend ES as the actions taken by enterprises for stakeholder’s satisfaction without compromising on standards related to environmental protection. But in the context of the business world, the notion of ES belongs to a vast horizon ranging from environmental aspects (Nidheesh and Kumar, 2019), social aspects (Doğu and Aras, 2019), and economic aspects (He et al., 2020) that is also regarded as triple bottom line (TBL) effect. There is a paradigm shift in business strategies related to environmental sustainability as the earlier version of ES was to control the rate of pollution that is now replaced with absolute ecological methods to be adopted by the businesses during their operations (Nassani et al., 2019). As a result of all these activities, the notion of ES has registered itself as a critical factor for safe and healthy business activities (Sandrin et al., 2018).

Meanwhile, scholars have also taped corporate social responsibility (CSR) with environment-related issues. For example, the studies of Kraus et al. (2020) and Ahmad et al. (2021b) are some ready examples. Though the concept of CSR has been part of academic discussion for many decades, this is quite recent that it has been linked with environment-related issues to have a better and sustainable future. It has been mentioned in the literature repeatedly that the hope to have a sustainable future will be possible when CSR is considered equally important to achieve economic, social, and environmental objectives by the businesses. For example, it is not possible to think about a sustainable future without the responsible behavior of employees (Murtaza et al., 2021). Unfortunately, in the context of developing countries, CSR has not been widely linked to improve environmental sustainability. The studies of Prasad et al. (2019) and Kong et al. (2021) are some ready examples that highlighted this negligence that sustainability orientation is not considered a priority in developing nations.

It is an acknowledging fact that the issue of ES is a new business language that pursues the firms to enhance profitability on one hand and impact the environment positively on other hand (Severo et al., 2015; Qureshi et al., 2019). ES is not a new idea as the concept was first introduced during the summit of the United Nations Global Compact’s initiative back in 2004. The theme of this summit was to highlight the environmental and social governance issues to the overall business communities of the world (UN, 2004).

The Global Climate Risk Index has placed Pakistan in 5th position in the list of nations most susceptible to climate change in its 2020 annual report. Pakistan lost the lives of 9,989 people, incurred economic losses of $ 3.8 billion, and faced 152 shocking climates between 1999 and 2018. Based on this information, it is established that Pakistan’s susceptibility to climate change is growing than ever before (Germanwatch, 2020). Pollution from the manufacturing sector is one of the biggest problems in Pakistan. Industrial pollution is particularly damaging to human health and the environment. In addition to automobile emissions, that account for 45% of air pollution, industrial pollution is a major cause of environmental degradation in Pakistan (Siddique and Kiani, 2020). Pakistan’s industrial segment is widespread, including chemicals, electrical goods, plastic industry, textile, fertilizers, and other products, base metals, non-metals, cement, automobiles, and light/heavy engineering. These production processes generate harmful emissions, air pollution, harmful fumes, and specks of dust (Mahmood et al., 2020). Unfortunately, there is little understanding of pollution prevention and improving pollution control in industry, especially in the manufacturing sector of the country. Current socio-environmental indicators and other related figures are worrying and unexpected. Pakistan is one of the 12 countries in Asia, with a total of 15 countries with high levels of industrial pollution. Every year, more than six and a half million people are hospitalized due to pollution caused by industrial operations (Ajmal, 2020). These figures paint a bleak picture of the future and calls for emergency measures to be taken especially in the industrial sector to mitigate the intensity of environmental degradation. Likewise, different international bodies striving for environment preservation throughout the globe have acknowledged the fact that only focusing on the cost associated with environmental discourse won’t appeal to policymakers sitting in their corporate offices to proactively respond to environmental issues (Seles et al., 2019). It has been witnessed that the majority of organizations react to environmental issues in order to meet the legal requirement imposed by the laws of a country (Guerci et al., 2016). For business leaders, the only motivator is their concern for profitability. In other words, businesses can be motivated to invest proactively in environmental management practices if they are believed that such investment assures a better payoff in long run. While, developed nations such as the United States and nations of the EU have remarkably raised their environmental standards, as they invest a lot, to preserve nature, the biggest problem lies within the developing and emerging countries in Asia and Africa, where environmental standards are far behind as compared to the developed nations (Andreosso O’callaghan et al., 2020; Huang and Iskandar, 2020). Moreover, the general belief, mostly in developing nations, is that investing in environmental initiatives involves huge costs that outweigh the benefits (Omisore, 2018). We argue that this belief is mistaken as studies have constantly reported that investing in environmental initiatives not only improves the environmental footprint of an organization but also improves its economic health in long run (Pham et al., 2021). To further aggravate the situation, the environment-related knowledge at the level of individuals is also poor in such countries, as in the case of developed nations, especially in the countries of EU, the better individual knowledge has also played a significant role to make the behavior of businesses more responsible toward the nature and environment (Dakhan et al., 2020). Given that the bottom line objectives will remain the sole priority of any business, the basic objective of the current study is to investigate the relationship between ES, economic performance (EP), and the environmental performance (ENP). The study also proposes environmental innovation (EI) as a mediator in the above proposed relations.

The contribution of the present study to extant literature is threefold. First, the present study aims at investigating the impact of ES on ENP and EP in a single model, whereas, the previous studies have largely linked ES with EP (Barba-Sánchez and Atienza-Sahuquillo, 2016; Gatimbu et al., 2018; Taliento et al., 2019; Adedoyin et al., 2020) or ENP (Radu and Francoeur, 2017; Haque and Ntim, 2018; Latan et al., 2018). Limited attention by previous researchers has been paid to the combined effect of ES on EP and ENP. To bridge this gap, we in the present study, aim to investigate the impact of ES on ENP and EP. Further, the findings of the current study may be helpful for the businesses in Pakistan in improving this mistaken belief that investing in eco-friendly initiatives involves additional costs which undermine the economic efficiency of a business. Second, extant researchers have long established that the relationship between ES on ENP and ES on EP is better explained in the presence of mediators and moderators (Chuang and Huang, 2018; Abbas, 2020; Kraus et al., 2020; Shang et al., 2020), we in this connection, argue that EI may be a potential mediator amongst ES to ENP and ES to EP. Third, the majority of previous studies related to ES are conducted in developed countries (Cheon et al., 2017; Wijethilake, 2017; Taliento et al., 2019; do Prado et al., 2020; Solarin and Bello, 2020), we pose that there is a difference between developed countries and emerging countries as emerging countries have different infrastructures, capital structures, regulations and perceptions about sustainable environment. Hence, generalizing the results from developed countries on emerging economies is not logical, therefore the present study tests the above-proposed relationships in the context of an emerging economy such as Pakistan, where the topic of the present study still lacks considerable attention. The remainder of this article is organized as follows, the coming section deals related literature and hypotheses development followed by the methodology section in which a discussion about population, sample, and instrument is given. Then comes the analysis section in which we have performed several statistical tests to validate our model and to evaluate our hypotheses. The last section deals with the discussion segment in which we have discussed our results concerning the previous researchers along with the implications and conclusions.

## Literature and Hypotheses

In the present ere, businesses all over the world have shifted their environmental policy from controlling pollution level to absolute prevention level as a result of different pressure groups who continuously exert pressure on organizations to operate in an eco-friendly manner (DeBoe, 2020). These pressure groups include different stakeholders for example NGOs, government, and different international bodies. Moreover, the psyche of stakeholders such as consumers and employees is also changing in response to different climate-related problems. Therefore, these stakeholders (consumers and employees) also expect businesses to adopt sustainable practices (Ahmad et al., 2021a; Amrutha and Geetha, 2021). Moreover, various researchers propose that adopting ES policy may actually lead an organization toward better EP (Tomšič et al., 2015; Schaltegger and Wagner, 2017). In other words, there exists a positive relationship between ES strategies and firm EP (Bilan et al., 2020). Organizations in the present competitive environment, adopt ES strategies not only to fulfill formal legal obligations but to satisfy different stakeholders, which in turn improves the overall competitive position of the organization and its EP (Caldera et al., 2018). Gatimbu et al. (2018) argued that the overall business performance of an organization is significantly affected by the organizational efforts to sustain the natural environment, which focuses on improving overall business efficiency through the reduction in cost, material wastages, and improved production technologies. Similarly, an organization can place itself in a higher position in competition by opting ES strategies that improve not only internal organizational processes but also external outcomes in the shape of enhanced sales and marketing results (Taherdangkoo et al., 2019). An effective response to ES strategies has multi-faceted results for an organization, which include social benefits (Karji et al., 2019), environmental benefits (Nidheesh and Kumar, 2019), and economic benefits (Niaki et al., 2019; da Costa Maynard et al., 2020). There is significant proof in the extant literature that verifies the relationship between ES and EP (Schaltegger and Wagner, 2017; Taliento et al., 2019; Alicia et al., 2020; Bilan et al., 2020). To sum, it is evident that pursuing ES policies better pays off an organization in the long run and helps the organization to gain a competitive advantage (Singh et al., 2019), reduce the cost of production, and the level of risk (Miemczyk and Luzzini, 2019), improves synergic impact (Pedercini et al., 2019), and fortifies overall organizational reputation (González-Rodríguez et al., 2019). Hence we propose:

***H_1__:_*** *Environmental sustainability is positively associated with economic performance.*

Generally, the term ES is associated with the efforts of businesses to preserve the natural environment. A strong ES orientation specifies the higher level of a firm’s commitment to proactively respond to ES practices to enhance firm efficiency, particularly, in the context of socially responsible behavior (Singh et al., 2019). Extant literature proposes that the engagement of a firm into ES practices eventually leads a firm to a higher level of ENP (Dieste et al., 2019; Gölgeci et al., 2019). There is a stream of researchers who contend that there is a positive association between ES practices of a firm and its ENP capability (Cooper, 1999; Epstein et al., 2015; Elmagrhi et al., 2019). Hence ES generally improves the ENP of an organization via effective implementations of ES practices. The existing literature provides sufficient grounds for the positive association between ES and ENP as the organizations with a higher level of ES emphasizes more to adopting the latest production technologies in order to reduce material cost and resource wastages, which in turn improves EP of an organization (Inman and Green, 2018; Singh et al., 2019).

There are two facets of how ES practices will eventually lead an organization toward superior ENP. In the first place, ES is regarded as an internal enabler that motivates organizations to conduct business activities in an environment that protects nature and improves the firm’s competitive advantage (Haseeb et al., 2019). Organizations with a higher level of environmental commitment willingly implement pollution prevention strategies (Awan et al., 2019), eco-friendly product designs (He et al., 2020), waste recycling and utilization (Jain et al., 2020). These ES actions eventually reduce the negative impact of a firm on a natural environment, which in turn improves the ENP of an organization. Additionally, the engagement of an organization in ES activities also improves employee productivity and commitment as they feel pride in serving an organization that works to protect the environment (Ma et al., 2020). As a result, the employees depict citizenship behavior that improves overall business efficiency, especially environmental efficiency (Luu, 2019).

In the second place, firms with a higher level of ES orientation are in a better position to build long-term relationships with consumers as consumers have respect for organizations that contribute positively to preserve nature (Norton et al., 2014; Hu et al., 2019). This perspective can also be seen in the study of Reyes-Menendez et al. (2018) who showed that an increase in online searches on the part of consumers about the social engagement and responsible practices of a business is evident of the fact that consumers prefer such organizations while they make purchase decisions. On a further level, the study of Reyes-Menendez et al. (2020) unveiled that consumers use positive word of mouth on different social media forums for responsible businesses to promote such brands among their peers and social circles. Hence ES engagement of an organization improves the overall image of the organization in the minds of customers, and they perceive the organization as a good corporate citizen, which produces ecology-friendly products that enhance the ENP of an organization. Hence it is proposed that:

***H_2_:*** *Environmental sustainability is positively associated with environmental performance.*

During the recent decade, there has been a growing concern on the topic of ES among academicians and policymakers around the globe, and this concern is a result of the increasing knowledge of different stakeholders about pollution prevention activities taken by the businesses. In response to all these, the companies are pushed to redefine their production processes in line with environmental protection policies (Radu and Francoeur, 2017). One possible way to deal with this challenging situation is the development of EI capability of an organization as the notion of EI introduces the concept of green product manufacturing (Belhadi et al., 2020), green administration (Singh et al., 2020), and green innovation practices during the process of new product development (Carrión-Flores and Innes, 2010). More specifically some scholars have stressed that innovation related to ecology not only induces the overall performance of an organization but also enhances its sustainable performance (Rehman et al., 2021). EI is organizational executions and variations concentrating on the environment, with inferences for corporations’ products, manufacturing processes, and marketing, with different degrees of novelty (Watson et al., 2018). Different researchers have also regarded EI as the green innovation in their studies and have poised that such practices eventually improve ENP capability and sustainable performance of a firm (Carrión-Flores and Innes, 2010; Long et al., 2017; Yu et al., 2017; Rehman et al., 2021). According to Ong et al. (2019), EI is a crucial function for improving the EP of an organization. Arguably, the activities pertinent to EI help an organization to meet regulatory requirements imposed by the state laws improves effective resource utilization, reduce duplication of resources, and reduces other environmental issues during the new product development phase via reduced emissions of toxic material and energy consumption (Costantini et al., 2017). Additionally, it is proposed by Singh et al. (2020) that EI activities induce ENP capability of an organization at each successive stage of production. Similarly, there are different researchers who acknowledged that EI enhances ENP of an organization through cleaner production (de Oliveira Neto et al., 2019), green product processes, and reduces material wastages (Susilawati and Kanowski, 2020), which provide a firm the opportunity of building a unique kind of competitive advantage over its rivals and hence induces ENP (Carrión-Flores and Innes, 2010; Long et al., 2017; Ong et al., 2019). According to Fernando and Wah (2017), EI practices significantly reduce the rate of carbon emission during the production process and improves efficient energy utilization. Similarly, there are different researchers (Zhang et al., 2017; Liao et al., 2018) who mentioned that EI is a useful tool that reduces production cost, enhances manufacturing innovation and corporate image, and eventually enhances ENP. Yenipazarli et al. (2019) also proposed that EI positively predicts ENP of an organization. All the above discussion leads us to propose:

***H_3_:*** *Environmental innovation is positively associated with environmental performance.*

***H_4_:*** *Environmental innovation mediates the relationship between environmental sustainability and environmental performance.*

The extant literature argues that there is a positive impact of EI on EP of an organization (Long et al., 2017). Different researchers poised that the higher level of a firm’s engagement in EI will lead toward better EP (Andries and Stephan, 2019), as through proactive ecological innovative strategies, the management can adopt excellent business measures that eventually enhance the overall EP of an organization (Tessitore et al., 2010; Boons et al., 2013). The steps taken by an organization to prevent pollution level facilitate an organization to reduce those element in the production level that are unhealthy for the environment in order to shrink life cycle impact on the environment and design products with lower life cycle cost which ultimately lowers down the substantial cost of production (Barbieri et al., 2020). In other words, a higher level of EI commitment may provide an opportunity to enhance overall business efficiency (Li, 2014). Additionally, firms can also have a competitive advantage in successive stages of product design and development, such as the reduction in product waste, recyclable design, and product maintenance. Hence the net result of environmental engagement is a significant reduction in production costs, which undoubtedly improves the economic health of an organization (Galeotti et al., 2020).

Alternatively, there is a strong possibility that the Environmental efforts of an organization will earn an excellent environmental reputation for a firm, and a firm this way may place itself into a position for charging a premium price and increased level of sales (Ponte, 2020). There is a long discussion among academicians and researchers on the relationship between ES and EP. Early research studies in this regard suggest that there is a trade-off between EI and EP, and the reason for that lies in the explanation that expenditures to carry out an EI may divert the attention of management and could raise the overall expenditures (Figge and Hahn, 2012; Saunders et al., 2020). But during the last two decades, there is a paradigm shift in this thinking as different researchers (Boons et al., 2013; Li, 2014; Long et al., 2017) showed that investing in innovation related to environmental activities can earn a win-win situation for a firm economically and environmentally in the long run. Investing in EI may enhance organizational competitiveness due to improved technical and technological efficiency and customer demand for eco-friendly products. Similarly, with the help of EI strategies, the firm can reduce material cost and waste disposal, and finally, a firm may be able to reduce pressure from different communities. These benefits, when looked into the perspective of cost-benefit analysis, outperform the cost incurred on EI. The natural-resource-based view also supports the notion that in the coming future, firms’ commitment related to EI will decide the economic and environmental destiny of a firm (De Stefano et al., 2016). Hence we propose the following hypotheses:

***H_5_:*** *Environmental innovation is positively associated with the economic performance.*

***H_6_:*** *Environmental innovation mediates the relationship between environmental sustainability and economic performance.*

## Materials and Methods

### Sample and Data Collection

The data for the present study were collected from manufacturing firms located in Lahore, Gujranwala, Karachi, and Sialkot cities of Pakistan. These manufacturing firms are related to a variety of industries, like the electronics industry, rubber and plastic industry, food processing industry, pharmaceuticals, chemicals, and so on. During the data collection procedure, we firstly selected different universities from various cities of Pakistan that were offering MBA degree programs for professionals. We then contacted the relevant staff and asked for their willingness to help in the data collection process for the present survey. After seeking their willingness, we provided them multiple copies of a printed version of the questionnaire which they distributed among students who, as a professional, were working in different manufacturing organizations. Before filling the questionnaire, the respondents were asked a screening question “whether your organization is involved in any EI and sustainability activity?” Those who answered positively were then asked to participate in the process of filling the questionnaire. We then collected the filled questionnaires from the staff members of concerned universities. We distributed 500 questionnaires among respective staff members of universities. Initially, we received 102 fully filled questionnaires, so in order to boost the response rate, we repeatedly followed up with the staff members who did not respond positively. With repeated followed-up efforts, the valid responses increased to 269, which means a response rate of 53.8%.

### Measures

We used existing scales developed by previous researchers for different variables. Thus the reliability and validity of the questionnaire is pre-established. For example, we adapted the items of ES from a reliable and valid scale on corporate sustainability, developed by Aktin and Gergin (2016). The construct of ES was comprised of eight items. Similarly, the scale of EP was adapted from Glaister et al. (2008) this scale consisted of five items. The scale of ENP was adapted from Melnyk et al. (2003) and Daily et al. (2007), this scale was comprised of seven items. Lastly, the items of EI were adapted from the study of Song and Yu (2018). This construct of EI was comprised of six items. We used a seven-point Likert scale to collect the responses.

## Results

### Analysis of the Data

Table 1 presents the results of respondents in terms of their experience, type of industry, and the number of employees. We divided the experience of respondents into four different categories ranging from 1 to 3 years to more than 12 years of experience. The highest category belongs to respondents who are having experience between 4 and 6 years as they contribute 36.06 percent. Whereas the lowest category in this regard is respondents having experience between 1 and 3 years of experience and one reason for this category to be ranked as lowest is that the sample data of the present study were collected mostly from manger level ranks, which includes more experienced people in most cases. Similarly, we received data from six types of industries included Rubber and Plastic, Electronics, Food Processing, Pharmaceutical industry, Textile and apparel, and others. The highest category of respondents in this regard belongs to the textile sector, which is one of the most significant contributors to the country’s GDP. In our data, this category comprises almost 32 percent of a total of 269 respondents, and the lowest category belongs to the others category, which only accounts for 5 percent of respondents. Finally, we have information of organizations in which respondents were working related to numbers of employees that a particular firm is possessing, according to the results, the data were collected from a mix of organizations including large organizations, medium and small organizations. For example, the respondents belonging to organizations that possessing employees less than 200 is the lowest category of respondents as only 7.81 percent data were collected from those organizations and the highest category in this regard includes organizations having < 5,000 employees as the employees responded from those organizations comprised almost 27 percent of our total sample of 269.

**TABLE 1:** Demographic profile of respondents.

**Description**	**Frequency**	**Percent**
**Experience**		
1–3	32	11.89
4–6	97	36.06
7–12	84	31.23
>12	56	20.82
**No. of Employees**		
<200	21	7.81
<500	27	10.00
<1,000	36	13.33
<2,000	64	23.79
<3,000	49	18.21
<5,000	72	26.76
**Type of industry**		
Plastic/Rubber	31	11.52
Textile and apparels	84	31.23
Food processing	41	15.24
Pharmaceutical	39	14.50
Electronics	29	10.78
Other	45	16.73

### Common Method Bias

According to Podsakoff and Organ (1986), when data are collected for the independent variable and dependent variable from the same individual, then there may exist the issue of common method bias. In other words, there are higher estimates than the actual estimates of the association between the constructs. In order to address the issue of common method bias, we performed a single factor analysis as recommended by Podsakoff and Organ (1986). The results showed that the single factor was explaining 39.4% of the total variance, which is indicative that there is the absence of single factor dominance. Similarly, the correlation analysis also revealed the absence of common method bias as if the issue of common method bias exists, and then correlation values should be close to 0.9, which is not the case in our data. By these findings, we are confident that our data is not suffering from the issue of common method bias.

In order to assess whether the model fits the data, we conducted confirmatory factor analysis (CFA) for our four constructs including ES, EI, EP, and ENP. Our initial CFA suffered from some problems such as weak loadings of some items to its respective constructs (we deleted one item of ES and ENP). We also drew correlations between some error terms to get a better model fit. After these adjustments, we finally received a better model fit (χ^2^/df = 2.68, CFI = 0.96, GFI = 0.93, IFI = 0.94, and RMSEA = 0.059). in this regard, we reported some important details in Table 2 related to model fit, means, standard deviation, correlation, reliability, validity, and multi-collinearity results. The results of Table 2 show sufficient values to accept reliability (as α and CR values > 0.7 for all cases) and validity results (as AVEs > 0.5 for all constructs). Likewise, we also reported multi-collinearity in Table 2, for this purpose we compared each construct’s square root of AVE value with correlation values. As the value of the square root of AVE for each construct is larger than the correlation values, hence it is established that there is less fear of multi-collinearity in our data.

**TABLE 2:** Correlations, validities, and reliabilities.

**Variables**	**Mean**	**SD**	**ES**	**EI**	**EP**	**ENP**	**α**	**CR**	**AVE**	**MSV**	**ASV**
**ES**	5.53	0.82	**(0.75)^a^**	0.531^**^	0.311^**^	0.168^**^	0.92	0.93	0.56	0.28	0.14
**EI**	5.41	0.92		**(0.81)^a^**	0.233^**^	0.257^**^	0.89	0.91	0.65	0.28	0.13
**EP**	5.95	0.98			**(0.73)^a^**	0.581^**^	0.91	0.92	0.53	0.34	0.148
**ENP**	5.84	0.85				**(0.76)^a^**	0.83	0.84	0.58	0.34	0.14
	(**χ^2^/*df*** = 2.68, **CFI** = 0.96, **GFI** = 0.93, **IFI** = 0.94, and **RMSEA** = 0.059)*^**^										
	^***^ Model fit indices for measurement model										
	*n* = 269										
	*p* < 0.001										

ES, Environmental sustainability; EI, environmental innovation; ENP, environmental performance; EP, economic performance; CR, composite reliability; AVE, average variance extracted; MSV, maximum shared variance; ASV, average shared variance; a, square root of AVE; SD, standard deviation. The bold values represent the square root of AVE for a construct. **, *** represent significant values.

*^a^The value of the square root of AVE is significant.*

### Hypotheses Testing

**FIGURE 1:**
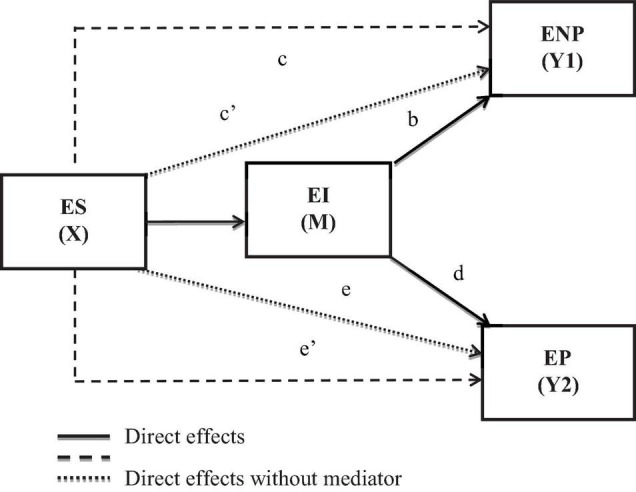
Proposed research model: ES (X), the independent variable; ENP (Yi), the dependent variable; EP (Y2), the dependent variable; EI (M), the mediating variable; C’, direct effect of X on Y1 with the effect of the mediator (M); e’, direct effect of X on Y2 with the effect of the mediator (M); C, direct effect of X on Y1 without mediator; e, direct effect of X on Y2 with mediator.

The results for hypotheses were drawn by using AMOS software, we performed structural equation modeling (SEM) three times as model 1, model 2, and model 3. In model 1, we observed direct results (ES→ENP = 0.58, *p* < 0.001; ES→EP = 0.79, *p* < 0.001; EI→ENP = 0.63, *p* < 0.001) which were positive and significant and hence provide sufficient grounds to accept H1, H2, H3, and H5. Moreover the results of model fit were also significant (χ^2^/*df* = 3.65, CFI = 0.89, GFI = 0.88, IFI = 0.90, and RMSEA = 0.068). In model 2, we reported mediation results to test our H6, for doing so, we performed bootstrapping for a larger sample of 5,000 in AMOS which produced significant results to prove that EI mediates between ES and EP (ES→EI→EP = 0.074^∗∗^, *p* < 0.001). It is worth noting that the model fit results are significantly improved as compared to model 1 (χ^2^/*df* = 2.81, CFI = 0.92, GFI = 0.90, IFI = 0.92, and RMSEA = 0.057) which is indicative that the variable EI, as a partial mediator, better explains the relationship of ES and EP. Lastly in model 3, we again tested the mediation results separately for our H4 using the same process as explained above for H6. The results of model three were supportive to accept H4 (ES→EI→ENP = 0.092^∗∗^, *p* < 0.001), furthermore, the results of model fit produced even better results in comparison to model 1 and model 2 which means that EI, as a mediator, is well suited between the relationship of ES and ENP. In a nutshell, the results proved that although the mediation results are supportive for both H4 and H6 but in the case of H4, these results are more suitable. The empirical results of hypotheses testing can be seen in Table 3. Figure 1, presents the research model of the current study.

**TABLE 3:** Hypotheses testing.

	**Path**	**Beta value**	**LLCI/ULCI**	**Supported/**
				**Not-supported**
**Model 1: Direct effects (H1, H2, H3, and H5)**				
	ES→ENP	0.58^**^	0.163/0.289	Supported
	ES→EP	0.79^**^	0.530/0.989	Supported
C	EI→ENP	0.63^**^	0.437/1.021	Supported
C	EI→EP	0.72^**^	0.492/0.882	Supported
(**χ^2^/*df*** = 3.65, **CFI** = 0.89, **GFI** = 0.88, **IFI** = 0.90, and **RMSEA** = 0.068)^***^				
**Model 2: Indirect effect (mediation model for H6)**				
	ES→EI→EP	0.074^**^	0.197/0.279	Supported
**χ^2^/*df*** = 2.81, **CFI** = 0.92, **GFI** = 0.90, **IFI** = 0.92, and **RMSEA** = 0.057)^***^				
**Model 3: Indirect effect (mediation model for H4)**				
	ES→EI→ENP	0.092^**^	0.183/0.201	Supported
**χ^2^/*df*** = 2.51, **CFI** = 0.94, **GFI** = 0.91, **IFI** = 0.95, and **RMSEA** = 0.051)^***^				

**Significant β-value; ***significant model fit values.

## Discussion

The present study has some important contributions for extant literature in the context of ES, for example, the findings of our study prove that proactive response from organizations to address the issue of ES has important outcomes in the context of overall business excellence. Similarly, the adaptation of ES strategies into the operations of a firm takes a firm toward internationally accepted standards relating to ES. This finding also receives support from extant researchers (Delmas and Blass, 2010; Spencer et al., 2013; Caldera et al., 2018; Danso et al., 2019; Adedoyin et al., 2020). Hence it is essential for the business to engage in ES practices in order to enhance overall business performance. Engagement in the activities of ES is also important for organizations as in the present era the notion of ES is receiving a lot of attention from various stakeholders including customers. It is worthwhile to mention here that the engagement of a firm in ES activities is particularly important in a developing economy context, such as Pakistan’s economy, in order to receive significant structural support from stakeholders to lessen institutional structures and eventually improve business performance.

Similarly, another important finding of the present study is to acknowledge the fact that ES improves ENP and EP of an organization significantly, as indicated by the empirical results of the present study. This finding is also in line with previous studies (Barba-Sánchez and Atienza-Sahuquillo, 2016; Cheon et al., 2017; Adedoyin et al., 2020; DeBoe, 2020). One of the major objectives of the present study was to examine the impact of EI activities as a mediator between ES to ENP and ES to EP. In this context, the findings of the present study confirm that EI mediates between both ENP and EP. This finding also receives support from existing literature (Costantini et al., 2017; Fernando and Wah, 2017; Long et al., 2017; Ong et al., 2019; Singh et al., 2020). EI can minimize environmental degradation by developing products that are eco-friendly in nature. Hence EI can lead an organization toward better energy usage, reduce resource wastages, and improves overall business excellence in the long run. Eco-Innovation in production processes and design improves the ENP and the overall EP of manufacturers. Given the consensus that EI can boost economic and environmental growth, our empirical findings confirm that EI commitment of manufacturing firms can bring long-term sustainable performance. Therefore in light of growing concerns about climate change and sustainable development, understanding when and why firms’ EI impacts their performance is important. It can help to engage more firms in EI activities and to design policies that support firms in doing so. On a final note, the finding of the current study will also be helpful in negating this mistaken belief that environmental investment undermines the economic efficiency of an organization, as the results of our study have proved that ES not only improves ENP but also enhances the economic efficiency of a business in the long run.

### Implications

The current study offers some significant theoretical and practical implications. We discuss some theoretical implications in the first place. In this regard, our study adds to the available literature on sustainability from the perspectives of both; EP and ENP. Prior literature has mostly investigated either the economic perspective (Gatimbu et al., 2018) or the environmental perspective (Repar et al., 2017). Therefore, the current study is an important contribution as it considered not only the economic aspect but also the environmental aspect. Specifically, our study also adds to the theory of sustainability as the current study unveils that by focusing on the ecological dependency of economic and social systems, ES illuminates the mutual effects between environmental degradation caused by human activities and the perils to human systems presented by global environmental problems.

In the second place, our study also offers some practical implications. For example, our study is one of those pioneering studies that bring it to the fore for the policymakers that participation in eco-friendly initiatives not only improves the environmental footprint of an organization but also improves the bottom line (economic) performance of an organization. Moreover, by engaging in sustainable practices, an organization is likely to develop a solid base of competitive advantage over its rivals as the eco-friendly innovations reduce the production cost, on one hand, whereas on other hand, such moves also enable an organization to produce value addition in a manner that is not easy to imitate by the rivals. Yet another important implication of our study lies in improving the environmental psychology of the policymakers of different businesses to assume ES as an added cost that outweighs the benefits (especially the economic benefits) is misleading.

### Limitations

The present study also has some limitations, but we believe that these limitations open new horizons for future researchers. First, this study used a cross-sectional survey design to collect data and is thus limited to a particular time of measurement. Hence, it doesn’t help to determine the cause and effect relationship in a meaningful manner. It is also susceptible to bias due to low response and misclassification due to recall bias, hence future researchers are required to apply longitudinal data. Second, the validity of this research is limited due to the difficulty in data collection. This research did not take a random sample, instead, the sample was drawn from institutions with which the authors had an existing relationship. Moreover, not all organizations responded to the questionnaires sent. These factors might affect or limit the validity of this write-up. So the future studies must address this issue by taking large and more diverse samples. The third limitation is the geographical context of data collection as most data were collected from the specific geography and most respondents were belonging to Punjab province, due to this issue, the results of the present study may not be generalized to other regions with confidence. In this context, future researchers are required to prepare a data set that represents diversity in terms of geography and segments in order to better represent the whole population and thus generalizing the results for the entire population with confidence. Similarly, our findings are based on data from Pakistani firms only. Although the conflicting nature of economic and environmental sustainability is a particularly prominent issue for emerging economies, further studies involving cross-national comparisons of different countries would increase the generalizability of the findings. Lastly, the current study has missed to include environmental factors especially environmental CSR and environmental strategy into the current model of the study. We think including such variables in the current model may also be interesting for future studies.

## Conclusion

Global warming, pollution, environmental issues, and diminishing resources have forced nations throughout the globe to pay higher attention to the issue of environmental sustainability. In this regard, ES and EI have become a preferred choice for businesses today in order to gain a competitive advantage and address different pressure groups in an efficient way. In this context, technological progress and innovation have emerged as critical factors responsible for business development. It is clear that eco-innovations and developments are the way forward for the manufacturing sector. The manufacturing sector contributes significantly to the economic development of Pakistan. However, considering the shortage of resources, they ignore advancement in technology and social welfare measures as they compete at low cost. Ultimately, sometimes they ignore regulatory authorities’ stringent norms regarding, pollution prevention.

In the light of our findings, Pakistani manufacturing sector should give priority to waste reduction, green manufacturing, healthy organizational culture, social welfare, appropriate disposition of waste. Climate change continues to have a rapid impact on the world and especially for Pakistan, its results are horrible. Countries are taking a series of measures to combat climate change. At this point, the environmental impacts of manufacturing organizations should not be ignored, especially in countries like Pakistan that is already vulnerable to climate change impacts. In this regard, it is notable that there exists a lack of commitment from businesses to proactively participate in ES practices as the majority of organizations just respond to ES in order to meet the regulatory requirement. This is high time to change this attitude to preserve nature. The results of the present study may provide motivations to policymakers to search for ways to implement environmental laws in a more preferred format. Arguably, our results will be eye-opening for the manufacturing sector of Pakistan to understand that engaging in sustainable practices is in the own interest of this sector because such engagement improves economic efficiency. Moreover, by embracing sustainability as a new business language, the manufacturing organizations of Pakistan can also earn a good reputation as contemporary consumers show respect for the corporations that adopt sustainable practices.

## Data Availability Statement

The data will be made available on a reasonable request from the corresponding author(s).

## Ethics Statement

Ethical review and approval was not required for the study on human participants in accordance with the local legislation and institutional requirements. The patients/participants provided their written informed consent to participate in this study.

## Author Contributions

All authors listed have made a substantial, direct and intellectual contribution to the work, and approved it for publication.

## Conflict of Interest

The authors declare that the research was conducted in the absence of any commercial or financial relationships that could be construed as a potential conflict of interest.

## Publisher’s Note

All claims expressed in this article are solely those of the authors and do not necessarily represent those of their affiliated organizations, or those of the publisher, the editors and the reviewers. Any product that may be evaluated in this article, or claim that may be made by its manufacturer, is not guaranteed or endorsed by the publisher.
